# The combination therapy of subtenon triamcinolone acetonide injection and intravitreal brolucizumab for brolucizumab-related intraocular inflammation

**DOI:** 10.1097/MD.0000000000027580

**Published:** 2021-10-22

**Authors:** Yumi Shigemoto, Yoichi Sakurada, Yoshiko Fukuda, Mio Matsubara, Ravi Parikh, Kenji Kashiwagi

**Affiliations:** a Department of Ophthalmology, Faculty of Medicine, University of Yamanashi, Japan; b Manhattan Retina and Eye Consultants, New York, NY; c New York University Grossman School of Medicine, New York, NY.

**Keywords:** age related macular degeneration, brolucizumab, intraocular inflammation, subtenon triamcinolone acetonide

## Abstract

**Rationale:**

Brolucizumab is a novel anti-vascular endothelial growth factor agent with clinical trials demonstrating excellent efficacy for neovascular age-related macular degeneration (AMD) in both visual and anatomic outcomes. However, there is concern of intraocular inflammation (IOI), and we propose concurrent subtenon triamcinolone acetonide (STTA) to prevent IOI.

**Patient concern:**

A 73-year-old man was treated with aflibercept for neovascular AMD in his right eye. Despite 11 months of monthly intravitreal aflibercept injections, optical coherence tomography demonstrated persistent exudation. Ten days following his second brolucizumab injection, the patient presented with decreased vision due to vitritis in his right eye

**Diagnosis:**

Brolucizumab-related IOI in neovascular AMD refractory to aflibercept.

**Interventions:**

A combination therapy involving of intravitreal brolucizumab and STTA

**Outcomes:**

The anti-vascular endothelial growth factor inhibitor was changed back to aflibercept; however, exudation persisted. Therefore, a combination therapy involving STTA (5 mg/0.5 mL) and intravitreal injection of brolucizumab (6.0 mg/0.05 mL) was performed to treat the exudation and as prophylaxis to recurrent IOI. Combination therapy achieved no recurrent IOI and resolution of exudation with 8-week treatment intervals.

**Lessons:**

This case might indicate that STTA is not only an optimal treatment option for brolucizumab-related IOI but also a preventive agent for this condition.

## 1 Introduction

Age-related macular degeneration (AMD) is a neovascular and inflammatory disease attributed to a multifactorial combination of genetic and environmental factors.^[1]^ To date, regular intravitreal injection of vascular endothelial growth factor (VEGF) inhibitors have been the first-line treatment option for neovascular AMD to both stabilize and improve vision.^[2–4]^ However, frequent injections are a substantial burden to both patients and health care providers. Several clinical trials have attempted to reduce the number of injections using the *pro re nata* and treat-and-extend regimens.^[5,6]^ Brolucizumab is a novel VEGF inhibitor approved by the US Food and Drug Administration on October 2, 2019. Brolucizumab is the smallest known VEGF inhibitor with a molecular mass of 26 kDa. Notably, its binding capacity to VEGF is approximately 11 times higher compared to aflibercept.^[7]^ In a phase 3 HAWK/HARRIER study, brolucizumab showed a longer durability compared with aflibercept.^[8]^ However, following real-world use of brolucizumab numerous reports of intraocular inflammation (IOI) were reported leading to many physicians avoiding its use as sterile IOI must often be treated as infectious endophthalmitis, a devastating complication of intravitreal injections. Once IOI is diagnosed, systemic and/or topical corticosteroid therapy are effective treatment options.^[9–11]^ Subtenon triamcinolone acetonide (STTA) injection is 1 potential treatment for IOI, however, no studies have reported whether this treatment has a potential prophylactic effect on brolucizumab-related IOI. In this case study, we report the potential preventive effect of STTA on brolucizumab-related IOI.

## 2 Case report

**Figure 1:**
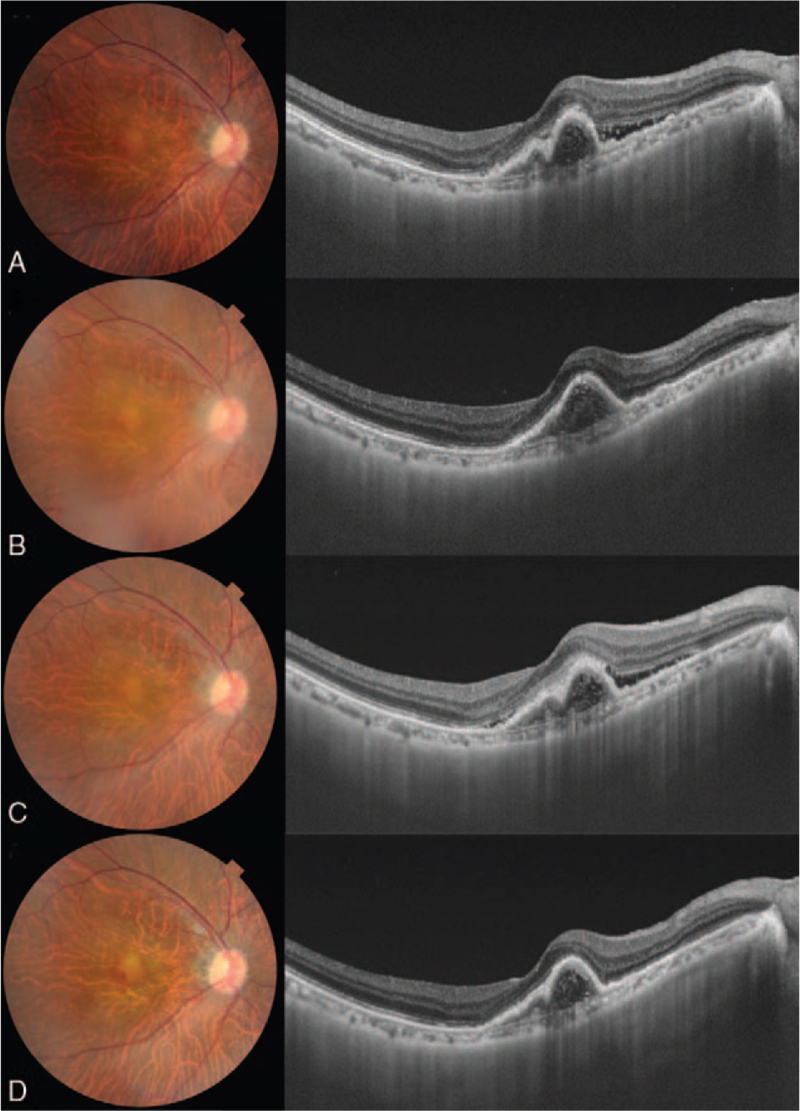
A 71-year-old male with type 1 macular neovascularization secondary to age-related macular degeneration treated with aflibercept injection in the right eye. (A) A horizontal OCT scan through the right macula shows residual subretinal fluid 1 month after the 12^th^ intravitreal aflibercept injection. The BCVA was 0.6 in the right eye. (B) Ten days after the 1^st^ brolucizumab administration, a color fundus photograph shows vitreous opacity resembling fog. The horizontal OCT scan shows a dry macula. The BCVA was maintained at 0.6 in the right eye. (C) One month after switching back to aflibercept, the horizontal OCT scan demonstrates residual subretinal fluid. The BCVA was 0.5 in the right eye. (D) One month after the combination therapy involving brolucizumab and subtenon triamcinolone acetonide, the horizontal OCT scan demonstrates resolution of macular exudation. The BCVA was 0.6 in the right eye. BCVA = best-corrected visual acuity, OCT = optical coherence tomography.

A 71-year-old male patient initially presented with a complaint of visual deterioration in his left eye. The decimal best-corrected visual acuity (BCVA) in his left eye was 0.7. His left eye was diagnosed with type 1 macular neovascularization secondary to neovascular AMD. Despite monthly intravitreal injection of aflibercept (2.0 mg/0.05 mL), the BCVA deteriorated from 0.7 to 0.2 in the left eye during a 24-month follow-up period. Two years after the initial onset, the patient complained of visual deterioration in his right eye. A comprehensive examination revealed that type 1 neovascularization secondary to neovascular AMD was also present in the right eye. The eye was also treated with aflibercept following *pro re nata* regimen; however, monthly injections were performed for a period of 11 months with no resolution of exudation in his right eye. Due to the inability for aflibercept to resolve his exudation and previously declining vision in his left eye, brolucizumab (6.0 mg/0.05 mL) was initiated in his right eye. Although 3 monthly intravitreal injections of brolucizumab were scheduled, the patient complained of blurred vision, which was secondary to vitritis from IOI 10 days after the 2^nd^ brolucizumab injection. It is important to rule out infectious endophthalmitis related to intravitreal administration. The patient had no previous history of uveitis and brolucizumab-related IOI was frequently seen 1 to 2 weeks after intravitreal brolucizumab administration. Therefore, brolucizumab-related IOI was diagnosed in the right eye. STTA (5 mg/0.5 mL) was administered in that eye. Four weeks after STTA, vitritis resolved, and the patient's symptoms were relieved. Nevertheless, recurrent exudation (which would lead to irreversible blindness if left untreated) was observed 4 weeks after STTA administration. Thus, treatment with aflibercept was resumed; however, a single intravitreal injection of aflibercept did not achieve complete resolution of exudation in right eye. At that point, the BCVA was 0.5 and 0.15 in the right and left eye, respectively, the right eye was the dominant eye. To address his vision threatening exudation and to prevent the adverse event of IOI we proposed a combination therapy involving concurrent intravitreal brolucizumab (6.0 mg/0.05 mL) and STTA (5 mg/0.5 mL) as a treatment option for neovascular AMD refractory to aflibercept with a history of brolucizumab-related IOI. The regimen was initiated upon the patient's consent. No inflammation or exudation was observed at 4 weeks after the 1^st^ combination therapy. The BCVA was maintained at 0.6 in the right eye; however, a recurrent exudation was seen on optical coherence tomography (OCT) 8 weeks after the combination therapy. Therefore, the 2^nd^ combination therapy was repeated. Although the combination therapy was administered 3 times because a recurrent exudation was seen on OCT, neither IOI nor intraocular pressure (IOP) elevation was observed. A total of 3 times combination treatments of STTA and intravitreal brolucizumab were administered over 16 weeks. The decimal BCVA in the right eye was retained at 0.6, 16 weeks after switching to the combination therapy. Figure 1 shows color fundus photography and OCT images of the patient over the time course. In this patient, brolucizumab appears to have a superior anatomic outcome in resolving exudation than aflibercept. Further the STTA (a treatment for IOI) may have had a prophylactic effect of preventing recurrent IOI. The combination of STTA and intravitreal brolucizumab was successful resolving exudation if given every 8 weeks while also preventing any recurrence of IOI.

## 3 Discussion

In the current report, the patient's eye with neovascular AMD exhibited brolucizumab-related IOI, including vitritis. After a reduction in inflammation after the administration of STTA, combination therapy involving brolucizumab and STTA was utilized because exudation had recurred. However, thereafter, IOI did not recur, and exudation was controlled with the repeated combination therapy.

Neovascular AMD is characterized by vision threatening exudation secondary from macular neovascularization in the elderly. Data from both clinical trials and real-world studies demonstrate the benefit of prompt and timely initiation of intravitreal anti-VEGF agents to control exudation.^[2,3]^ Brolucizumab is a novel commercially available VEGF inhibitor. Its advantage over prior agents is a greater binding capacity to VEGF compared with aflibercept, which leads to improved resolution of exudation.^[12]^ Several studies reported that brolucizumab were effective for neovascular AMD refractory to aflibercept.^[13,14]^ However, serious adverse outcomes related to IOI have limited utilization of brolucizumab.^[10,11,15]^

In the phase 3 HAWK/HARRIER study, the incidence of IOI in the 6.0 mg brolucizumab-treated group (4.0%) was higher than that in the 2.0 mg aflibercept-treated group (1.0%).^[8]^ In this trial, most cases were diagnosed as mild to moderate IOI. The cause of IOI was unclear and is currently under investigation. Several possible causes have been proposed, such as the formation of antibodies, immune status, and prior administration of anti-VEGF agents.^[9,16]^

No clear guidelines regarding IOI management exist; however, intensive treatment with corticosteroids, including topical, subtenon, intravitreal, and systemic administration is recommended.^[9–11]^ STTA was mainly performed for eyes with uveitis to reduce the inflammation and macular edema. In a recent report, Hikichi strongly recommended that immediate steroid therapy, especially STTA, should be administrated for eyes with brolucizumab-related IOI.^[10]^

In this case, vitreous inflammation without retinal occlusion and sheathed retinal vein appeared 10 days after the 2^nd^ brolucizumab injection. Four weeks after STTA administration, the inflammation completely resolved; however, after returning to aflibercept treatment exudation was not full resolved, as the right eye was the better-seeing eye there was concern in not controlling persistent exudation after it led to decreasing vision in the left eye. Therefore, combination therapy was selected to both resolve exudation and prevent the brolucizumab-related IOI. As expected, at 8-week intervals both exudation and IOI did not recur.

It is well known that repeated STTA administration increases the risk of IOP elevation and cataract progression.^[17]^ In this case, IOP elevation was not observed, and the patient's right eye was pseudophakia. Physicians should consider these adverse events when administering STTA.

This study is not free from certain limitations. This is a single case report, and it is unknown whether STTA administration could have a preventive effect on brolucizumab-related IOI in all eyes. To confirm the results of the present report, more similar cases should be assessed, and early development of guidelines regarding brolucizumab-related IOI management is desirable.

In conclusion, STTA might not only be an effective treatment choice for brolucizumab-related IOI, but also a preventive option for this condition if one needs to continually administer brolucizumab to control macular exudation.

## Author contributions

**Conceptualization:** Yoichi Sakurada.

**Data curation:** Yumi Shigemoto, Yoshiko Fukuda, Mio Matsubara.

**Supervision:** Kenji Kashiwagi.

**Writing – original draft:** Yumi Shigemoto.

**Writing – review & editing:** Yoichi Sakurada, Yoshiko Fukuda, Mio Matsubara, Ravi Parikh, Kenji Kashiwagi.
